# Post-synthetic pillaring enhances metal–organic framework capacitance

**DOI:** 10.1038/s42004-022-00648-w

**Published:** 2022-03-04

**Authors:** Victoria Richards

**Affiliations:** Communications Chemistry, https://www.nature.com/commschem

## Abstract

Electrically conductive two-dimensional metal–organic frameworks have emerged as promising materials for electronic and energy storage devices, but their stacked nature offers limited accessibility to the framework pores. Now, pillaring a conductive 2D MOF is shown to enhance gravimetric capacitance by more than double.

Electrically conductive metal*–*organic frameworks (MOFs) have been widely investigated in recent years thanks to the opportunities that combining framework conductivity with material porosity offers. Most electrically conductive MOFs reported so far are however two-dimensional in nature owing to favourable stacking interactions between conjugated ligands. This stacking between sheets limits ion accessibility to the internal pores, leading to inefficient diffusion. Now, Jihye Park and colleagues from the University of Colorado Boulder in the US demonstrate that post-synthetic pillaring of 2D MOFs is an effective strategy to produce conductive 3D frameworks with accessible pores (10.1021/acsnano.1c10838)^1^.

The researchers exploit copper nodes that initially adopt a square-planar geometry when combined with tetrahydroxy-1,4-benzoquinone (THQ) ligands^2^, but can post-synthetically accommodate 4,4’-bipyridyl pillar ligands in an octahedral coordination sphere (Fig. 1).

**Fig. 1:**
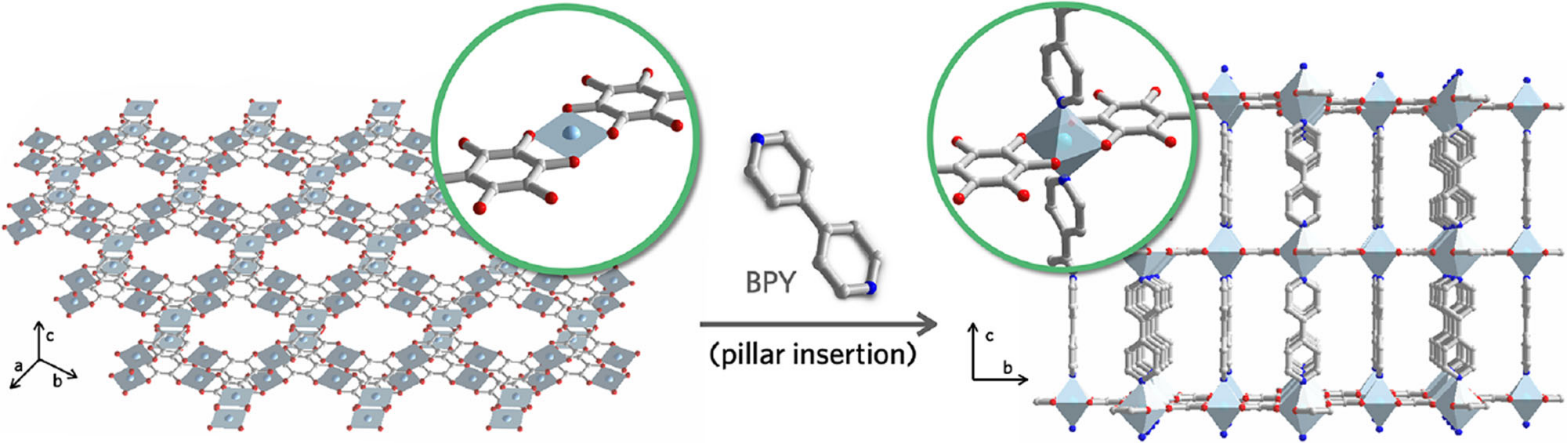
Pillaring strategy. Two-dimensional sheets of Cu–THQ are post-synthetically pillared with 4,4′-bipyridyl ligands to afford an electrically conductive three-dimensional framework. Grey: carbon; red: oxygen; light blue: copper; darker blue: nitrogen. Adapted with permission from *ACS Nano* DOI: 10.1021/acsnano.1c10838. Copyright (2022) American Chemical Society.

“We hypothesized that if the 2D sheet-stacked structure could be interrupted, we would gain insight into the structure–transport relationship”, comments Park. “Unlike post-synthetic pillar insertion demonstrated in conventional MOFs, we expected pillar insertion in conductive MOFs to serve as a chemical tool to manipulate charge transport pathways.” The team found that an increased interlayer distance between the sheets in the pillared MOF as compared with its 2D counterpart led to a decrease in bulk electrical conductivity. More encouragingly, however, electrochemical studies found the 3D MOF to display a gravimetric capacitance of 66.1 F g^−1^ at 10 mV s^−1^, more than double that of pristine Cu–THQ. The researchers attributed this to the increased electrochemical surface area that results from enhanced ion accessibility to the framework pores.

“Beyond our chosen platform, we expect pillar insertion to be a facile means to structurally diversify and chemically functionalize other existing 2D electrically conducting MOFs”, concludes Park. Indeed, when one considers the large pool of possible pillaring ligands and the increasing number of known conductive 2D MOFs, it is easy to imagine that opportunities to exploit this strategy will be numerous.
